# KCD: A prediction web server of knowledge‐based circular dichroism

**DOI:** 10.1002/pro.4967

**Published:** 2024-03-27

**Authors:** Damián Jacinto‐Méndez, Carmen Giovana Granados‐Ramírez, Mauricio D. Carbajal‐Tinoco

**Affiliations:** ^1^ Departamento de Física Centro de Investigación y de Estudios Avanzados del IPN Mexico City Mexico; ^2^ Facultad de Ciencias Matemáticas y Naturales Universidad Distrital Francisco José de Caldas Bogotá D.C. Colombia

**Keywords:** circular dichroism, protein structure, spectral prediction, web server

## Abstract

We present a web server that predicts the far‐UV circular dichroism (CD) spectra of proteins by utilizing their three‐dimensional (3D) structures from the Protein Data Bank (PDB). The main algorithm is based on the classical theory of optical activity together with a set of atomic complex polarizabilities, which are obtained from the analysis of a series of synchrotron radiation CD spectra and their related 3D structures from the PDB. The results of our knowledge‐based CD method (KCD) are in good agreement with measured spectra that could include the effect of D‐amino acids. Our method also delivers some of the most accurate predictions, in comparison with the calculated spectra from well‐established models. Specifically, using a metric of closeness based on normalized absolute deviations between experimental and calculated spectra, the mean values for a series of 57 test proteins give the following figures for such models: 0.26 KCD, 0.27 PDBMD2CD, 0.30 SESCA, and 0.47 DichroCalc. From another point of view, it is worth mentioning the remarkable capabilities of the recent approaches based on artificial intelligence, which can precisely predict the native structure of proteins. The structure of proteins, however, is flexible and can be modified by a diversity of environmental factors such as interactions with other molecules, mechanical stresses, variations of temperature, pH, or ionic strength. Experimental CD spectra together with reliable predictions can be utilized to assess eventual secondary structural changes. A similar kind of evaluation can be done for the case of an incomplete protein structure that has been reconstructed by using different approaches. The KCD method can be freely accessed from: https://kcd.cinvestav.mx/.

## 1 INTRODUCTION

Circular dichroism (CD) is a molecular spectroscopy that involves the differential absorption for left‐ and right‐handed circularly polarized light. CD is a rapid method that has been extensively used to characterize the secondary structure, folding, and binding properties of proteins. Moreover, CD can distinguish enantiomers (Eun et al., 2020) and is also sensitive to conformational changes (Miranda et al., 2001). The characterization of such properties requires minor quantities of protein and their experimental conditions can be easily modified. CD is closely related to the corresponding difference in refractive indices and both properties constitute the optical activity (OA) that was first identified at the beginning of the 19th century (Yang, 1996) and has been under study up to the present time. The first experimental measurements of the OA of polypeptides and proteins were made about 60 years ago (Woody, 2015). Since then, CD has been used to estimate the secondary structure conformation by different methods (Greenfield, 2006) and even the analysis of a particular spectral point can provide useful structural information (Dang & Hirst, 2001). As a technique, CD has been steadily improving and some of the most accurate spectral data can be obtained through synchrotron radiation circular dichroism (SRCD) (Wallace, 2000). It can be noticed that upgraded benchtop CD devices also provide quality spectra (Xie et al., 2022). At this point, it has to be stressed that the full potential of an experimental spectrum can be reached only if there is an accurate connection with the spectrum calculated from its corresponding 3D structure.

In 1960, Tinoco mentioned the difficulty of finding a successful theory of OA (Tinoco & Prigogine, 1962). Despite noticeable advances, the problem is still not fully solved even using a combination of quantum mechanics (QM) and a machine learning protocol (Zhao et al., 2021). The prediction of the CD spectrum of a given protein from its corresponding 3D structure is a theoretical challenge that has been approached from different points of view that are briefly described in the following lines. The DichroCalc (DC) method (Bulheller & Hirst, 2009) was one of the first prediction web servers of CD spectra and it is founded on the matrix model, which is an approximate version of the full QM approach (Besley & Hirst, 1999). A more rigorous version of this scheme has been recently published (Rogers et al., 2022). From another perspective, models based on the statistical analysis of the spectral database also deliver sound predictions of CD spectra. Among them, we can highlight the PDB2CD method that was first introduced in 2017 (Mavridis & Janes, 2017), and its upgraded version, PDBMD2CD, dates from 2020 (Drew & Janes, 2020). Similar in nature, the most recent release of the SESCA model relies on a Bayesian analysis to estimate the protein secondary structure composition and its CD spectrum (Nagy & Grubmüller, 2021a). For comparison purposes, we make use of the latest available versions of each model, which are denoted as DC, PDB2CD, and SESCA, respectively. On the other hand, our knowledge‐based CD approach (KCD) is based on the classical electromagnetic theory of OA, which mainly depends on effective atomic polarizabilities that are obtained from the analysis of measured CD data (Granados‐Ramírez & Carbajal‐Tinoco, 2022).

## 2 MATERIALS AND METHODS

### 2.1 A knowledge‐based model of CD

In 1965, DeVoe presented a classical description of diverse optical properties that include ellipticity, optical rotation, and extinction coefficient (DeVoe, 1965). The DeVoe scheme consists of a series of coupled oscillators that are mainly described by complex polarizabilities. In a first approximation, such polarizabilities were modeled by means of Lorentzian functions, which allow to obtain moderately good results (Applequist et al., 1979; Uporov et al., 2015). A more promising approach consists of determining a set of complex polarizabilities from measured spectral data with no preestablished functional forms. Such polarizabilities can be either referred to whole residues (Granados‐Ramírez & Carbajal‐Tinoco, 2020) or individual atoms (Granados‐Ramírez & Carbajal‐Tinoco, 2022). Of these two possibilities, the main advantage of the model relying on atomic polarizabilities is the capability of full automation, as presented here.

Within the DeVoe scheme, the main contribution to the molar ellipticity $\left[\theta \right]$ comes from the second‐order term of a Taylor expansion and it becomes our working hypothesis, namely (Granados‐Ramírez & Carbajal‐Tinoco, 2022), θ=−∑i,jImαiαjCijGij, where the coefficients $C_{\mathrm{ij}}$ are defined as $C_{\mathrm{ij}}=K\left[\left(e_{i}\times e_{j}\right)\cdot \left(r_{j}-r_{i}\right)\right]/\lambda^{2}$ and $G_{\mathrm{ij}}=r_{\mathrm{ij}}^{-3}\left[e_{i}\cdot e_{j}-3\left(e_{i}\cdot e_{\mathrm{ij}}\right)\left(e_{j}\cdot e_{\mathrm{ij}}\right)\right]$ $\left(i\neq j\right)$ are the elements of the dipole–dipole interaction matrix. Here, $\alpha_{i}$ and $e_{i}$ are respectively the complex polarizability and the unit vector of the induced electric dipole moment at site *i*. Additionally, *K* is a constant equal to $1.426468\times 10^{26}$, λ is the wavelength of the incident beams, $r_{\mathrm{ij}}=|r_{i}-r_{j}|$ is the distance between two point dipoles located at the positions $r_{i}$ and $r_{j}$, with $e_{\mathrm{ij}}$ being the unit vector along the line connecting them. Of course, the molar CD Δϵ can be obtained from the molar ellipticity through the expression $\left[\theta \right]=3298.21\Delta \epsilon$.

Each atom a belonging to a protein requires an appropriate assignment for its corresponding polarizability $\alpha_{a}$. Such assignment not only depends on the specific type of atom but also on its surrounding environment, which includes chemically bonded atoms as well as the different kinds of secondary structures of the adjacent residues. From this perspective, we propose the use of 21 isotropic atomic polarizabilities $\alpha_{a}$ that are variants of the commonly found atoms: H, C, N, O, and S. The details of such selection can be consulted in Granados‐Ramírez and Carbajal‐Tinoco (2022). Additionally, we make the distinction between four groups of secondary structures: α‐helices, β‐strands, coils, and others (including turns, bridges, and so forth). All of them are classified employing the STRIDE algorithm (Frishman & Argos, 1995).

The main source of high‐quality spectroscopic data, especially SRCD spectra, is the Protein Circular Dichroism Data Bank (PCDDB) (Lees et al., 2006; Ramalli et al., 2022; Whitmore et al., 2017), which provides the experimental CD spectra that are used together with their corresponding 3D structures from the Protein Data Bank (PDB) (Berman et al., 2000), in order to extract the necessary information for our KCD model. The deconvolution procedure to determine the set of effective atomic polarizabilities $\left\{\alpha_{a}\right\}$ is briefly depicted in the following lines and a more detailed description can be found in Granados‐Ramírez and Carbajal‐Tinoco (2022). We first mention that such polarizabilities not only depend on the type of atom and its position in the protein, but also on subtler properties such as the surrounding environment that includes the atoms of both own and neighbor residues, which are characterized by a specific type of secondary structure.

For our purposes, we require a more general property defined as $\left[o\right]=-\sum_{i,j}\alpha_{i}\alpha_{j}C_{\mathrm{ij}}G_{\mathrm{ij}}$, which is known as the OA. It can be noted that the real part of $\left[o\right]$ can be obtained by means of a Kramers‐Kronig transform of $\left[\theta \right]\;$(Granados‐Ramírez & Carbajal‐Tinoco, 2022; Moscowitz, 1962). The first stage of our approach consists of determining the mean atomic polarizability $\left\langle \alpha_{p}\right\rangle$ for a given protein p, such that ${\left\langle \alpha_{p}\right\rangle }^{2}=-\left[o_{p}\right]/{\left\langle \sum_{i,j}C_{\mathrm{ij}}^{p}G_{\mathrm{ij}}^{p}\right\rangle }_{\Omega }$. Here, the subindex Ω denotes a rotational average that is intended to take into account the different positions of the protein with respect to the incident beam. The OA of protein p can also be expressed in terms of the atomic polarizabilities, that is, $\left[o_{p}\right]=-\sum_{a,a^{\prime }}\alpha_{a}\alpha_{a^{\prime }}{\left\langle C_{aa^{\prime }}^{p}G_{aa^{\prime }}^{p}\right\rangle }_{\Omega }$, where the sites *i* and *j* have been replaced by their corresponding types of atoms a and $a^{\prime }$. In their turn, the atomic polarizabilities can be obtained from the expression αa=∑pkapReαp+∑pkap′Imαp. Here, the coefficients $k_{\mathrm{ap}}$ and $k_{\mathrm{ap}}^{\prime }$ are real and positive, and are determined by using a learning algorithm based on the Monte Carlo method (Granados‐Ramírez & Carbajal‐Tinoco, 2022). Both the learning and evaluation processes require a reliable metric to assess the closeness between modeled and experimental spectra.

There are various approaches intended to evaluate the accuracy deviation between the predicted and the corresponding measured spectrum (Nagy & Grubmüller, 2021b). A first option could be the root mean squared deviation (Drew & Janes, 2020; Granados‐Ramírez & Carbajal‐Tinoco, 2020). A significant disadvantage of this metric has to do with the large amplitude differences between spectra, which is an intrinsic feature of CD spectra and is related to the secondary structure composition. Consequently, such differences lead to large variations of the root mean squared deviation. It is possible to overcome such inconvenience by making use of the normalized root mean squared deviation (NRMSD) (Granados‐Ramírez & Carbajal‐Tinoco, 2022; Mao et al., 1982) that is a relative deviation with respect to the experimental spectrum. However, the use of squares may have the effect of an undesired sensitivity to outlying points (Press et al., 2007). Taking into account the ideas of robust estimation (Press et al., 2007), we propose an evaluation through a metric based on the normalized absolute deviation (NAD), namely, $\hat{\Delta }\left[\theta \right]=\sum_{i}^{M}|\left[\theta_{i}^{\exp}\right]-\left[\theta_{i}^{\mathrm{mod}}\right]|/\sum_{i}^{M}|\left[\theta_{i}^{\exp}\right]|$. Here, $\left[\theta_{i}^{\exp}\right]$ and $\left[\theta_{i}^{\mathrm{mod}}\right]$ are the experimental and the modeled spectral points, respectively. The total number of data points is *M* and it corresponds to discrete ellipticity data on the wavelength interval of 188 nm ≤λ≤ 244 nm. The interpretation of the metric $\hat{\Delta }\left[\theta \right]$ is straightforward since, for example, a value of 0.1 represents a 10% deviation with respect to the experimental curve and 0 indicates a perfect matching.

It should be pointed out that the most relevant contribution to the resulting set of polarizabilities comes from the inclusion of additional proteins to the training set, which now consists of 120 nonhomologous proteins, in comparison with the original set of 37 proteins (Granados‐Ramírez & Carbajal‐Tinoco, 2022). As a result, the addition of 83 new polarizabilities has increased the accuracy of the model. By construction, the algorithm can receive further improvements with the inclusion of the polarizabilities of more proteins. The current base of proteins is denoted as *b* and can be divided into three groups, according to their secondary structure content, and the proteins belonging to such groups are presented and characterized in Tables 1, 2, 3. The resulting base set of atomic polarizabilities is denoted as $\left\{\alpha_{\mathrm{ab}}\right\}$.

**TABLE 1:** The first group of the list of proteins belonging to the learning base.

	Proteins with:	*α*‐helices	PDB‐ID ($\hat{\Delta }\left[\theta \right]$)	
1n5u (0.026)	1lin (0.013)	2cts (0.030)	7atj (0.086)	1une (0.026)
2y3z (0.011)	1qfe (0.017)	7tim (0.028)	1bn6 (0.033)	5cpa (0.079)
1ymb (0.121)	1trz (0.039)	1qhj (0.018)	2oar (0.071)	2a65 (0.029)
2nop (0.082)	1nkz (0.019)	1hda (0.039)	2cfq (0.017)	1j95 (0.062)
1hzx (0.014)	2nr9 (0.014)	1gai (0.022)	6dhm (0.052)	1xag (0.045)
1ado (0.014)	1hrc (0.041)	2j0x (0.018)	2dhq (0.020)	1a49 (0.074)
1t4b (0.031)	1t5s (0.031)	2ccm (0.015)	1gpb (0.026)	1ha7 (0.018)
1a6m (0.020)	1ies (0.023)			

*Note*: These proteins contain at least 30% of α‐helices. Each protein is identified with the corresponding PDB code and is characterized with the normalized absolute deviation ($\hat{\Delta }\left[\theta \right]$), which is a measure of closeness between modeled and experimental spectra (see text).

**TABLE 2:** The second group of proteins from the learning base.

	Proteins with:	*β*‐strands	PDB‐ID ($\hat{\Delta }\left[\theta \right]$)	
1mol (0.093)	1air (0.043)	1a7h (0.062)	1ha4 (0.082)	2bb2 (0.062)
1cbj (0.056)	1m8u (0.337)	1jbc (0.157)	1ecz (0.088)	1stp (0.262)
4gcr (0.136)	1ubi (0.021)	3rn3 (0.076)	1auj (0.114)	1ba7 (0.133)
1jpc (0.160)	1k9b (0.049)	3gny (0.214)	1xl4 (0.018)	3jqo (0.047)
1ova (0.021)	1qlp (0.019)	1wnh (0.033)	5dfr (0.038)	1hk9 (0.035)
1bn8 (0.036)	2psg (0.111)	1uyn (0.030)	1v9u (0.139)	2cga (0.064)
2pab (0.090)	1thw (0.120)	1elp (0.056)	2rhe (0.034)	1bd7 (0.034)
1hk0 (0.205)	2yxf (0.068)	1rav (0.303)	1kcw (0.116)	1kit (0.032)
1les (0.209)	1ofs (0.141)	1fep (0.122)	2e8d (0.071)	5cha (0.164)
1nqh (0.039)	3est (0.059)	1a0s (0.030)	1ku8 (0.126)	1fcp (0.079)
1igt (0.150)	1hc9 (0.138)	1hcb (0.185)		

*Note*: Such proteins have a minimum content of 30% of β‐structures. The proteins are identified with their PDB code and the proximity between modeled and experimental spectra is described through the normalized absolute deviation ($\hat{\Delta }\left[\theta \right]$).

**TABLE 3:** The third group of proteins of the learning base, which consists of proteins of varied kinds of secondary structures.

	Proteins with:	Varied struct.	PDB‐ID ($\hat{\Delta }\left[\theta \right]$)	
1rhs (0.017)	3pgk (0.030)	1cf3 (0.041)	1ppn (0.069)	194l (0.023)
1r0i (0.072)	2fdn (0.045)	2pjf (0.072)	3pmg (0.012)	1hnn (0.019)
1hml (0.038)	1fa2 (0.019)	2j51 (0.025)	1dgf (0.058)	1scd (0.042)
1a8e (0.021)	1ed9 (0.026)	1vjs (0.053)	3dni (0.035)	2sns (0.027)
1sfl (0.039)	1azu (0.060)	9wga (0.086)	5pti (0.122)	3njw (0.103)
4kyp (0.036)	1blf (0.030)	6y84 (0.044)	1dot (0.019)	2gel (0.023)

*Note*: The proteins are identified with the related PDB code and are characterized by means of the normalized absolute deviation ($\hat{\Delta }\left[\theta \right]$).

Within the KCD model, the calculation of the CD spectrum of a given protein *p* requires the determination of the most suitable set of 21 atomic polarizabilities $\left\{\alpha_{\mathrm{ap}}\right\}$, in agreement with its secondary structure content. Once established, the set of 21 complex polarizabilities is utilized through the whole protein, giving as a result a mean‐field‐like approach. Therefore, according to the above‐mentioned procedure (Granados‐Ramírez & Carbajal‐Tinoco, 2022), $\alpha_{\mathrm{ap}}=\sum_{g}c_{g}\alpha_{\mathrm{ag}}+\sum_{i}c_{\mathrm{bi}}\alpha_{\mathrm{abi}}$, where $\alpha_{\mathrm{ag}}$ are polarizabilities of groups of proteins, the coefficients $c_{g}$ and $c_{\mathrm{bi}}$ are weight constants used to evaluate structural proximity, and the index *i* is the label of the proteins from the base. The coefficients $c_{g}$ are constants of order 1, whereas the individual weight constants have a more relevant role and are defined as $c_{\mathrm{bi}}=100\exp\left[-50\left(\left|a_{i}-p_{a}|+|b_{i}-p_{b}|+|c_{i}-p_{c}|+|o_{i}-p_{o}\right|\right)\right]/c_{t}$. Here, $a_{i}$ ($p_{a}$), $b_{i}$ ($p_{b}$), $c_{i}$ ($p_{c}$), and $o_{i}$ ($p_{o}$) are the structural contents of protein *i* from the base (structural contents of protein *p*) of *α*‐helices, *β*‐strands, coils, and other structures, respectively. The constant $c_{t}$ is used for normalization purposes. In the case of atoms belonging to D‐amino acid residues, their corresponding polarizabilities $\left\{\alpha_{a*}\right\}$ are properly modeled by taking the opposite sign of the real part of the regular polarizabilities $\left\{\alpha_{a}\right\}\;$(Granados‐Ramírez & Carbajal‐Tinoco, 2022). The capabilities of the described model are evaluated in the next section.

### 2.2 Evaluation of the KCD model

An evaluation of the KCD model consists of a comparison between the normalized spectral results predicted by the following models: KCD, PDB2CD (Drew & Janes, 2020), SESCA (Nagy & Grubmüller, 2021a), and DC (Bulheller & Hirst, 2009). Here, the normalization constant is determined from the summation of the absolute values of the corresponding experimental points. For comparison purposes, in Table 4 we present a series of 57 proteins from the PCDDB repository that are identified as the test set. Although the repository contains 741 CD spectra, not all of them are useful for our purposes. For certain proteins, the corresponding spectrum has been repeated from few to many times. Some examples are here indicated with the number of repetitions in parenthesis, that is, sodium/potassium transporting ATPase (255), ion transport protein (55), BH1501 protein (47), nav ms full length (18), nav ms pore (18), translocate actin recruiting phosphoprotein (15), lysozyme (14), MEG‐14 (10), EGFP (5), E5 (5), bovine collagen type II (5), formate oxidase (5), presenilin (4), among others. Such repetitions limit the statistical analysis to a single useful spectrum for each one of these proteins. In a few cases, also excluded, the CD spectrum is somewhat noisy or inexistent for wavelengths smaller than ~195 nm (e.g., cglbn, immunoglobulin g1, and ameliogenins). In some other cases, there is no structural information (e.g., cglbn, immunoglobulin g1, and ameliogenins). Of course, we do not take into account the learning set of 120 spectra. Moreover, the test set list includes 48 proteins from the PDB as well as 9 structures predicted by the AlphaFold program (Jumper et al., 2021).

**TABLE 4:** A series of 57 proteins that are utilized as a test set.

	Proteins used	For testing	Protein‐ID ($\hat{\Delta }\left[\theta \right]$)	
2gif (0.108)	1k6j (0.284)	2wjn (0.091)	1nek (0.064)	3kdp (0.078)
1qi7 (0.194)	2kit (0.723)	1pcr (0.096)	5jhe (0.222)	2hav (0.074)
1rh5 (0.071)	1b8e (0.176)	3qlp (0.520)	2iwv (0.388)	1h2s (0.146)
2vdf (0.662)	2j58 (0.269)	1l7v (0.169)	2dyr (0.275)	1sr5 (0.255)
3q9t (0.127)	1kpk (0.195)	1mdu (0.105)	1be3 (0.027)	2j41 (0.129)
5a37 (0.296)	1q5u (0.974)	2ox0 (0.203)	3go0 (0.149)	1oki (0.122)
5hvx (0.161)	5hvd (0.085)	4v40 (0.314)	p69905 (0.152)	1ytq (0.167)
6tav (0.709)	p07148 (0.107)	2zxe (0.105)	2kpk (0.379)	2wcb (0.134)
2m9g (0.181)	2nwe (0.054)	1ax8 (0.129)	3qtk (0.649)	1wyy (0.087)
6lhm (0.204)	3n23 (0.111)	4p90 (0.236)	4p9o (0.223)	4f4l (0.174)
q9avb0 (0.366)	q9buz4 (0.659)	p62568 (0.104)	p10451 (0.890)	q6gx35 (0.784)
p49810 (0.065)	q9m0n8 (0.318)			

*Note*: The proteins are identified by using their PDB code, except for the following proteins that are modeled through the AlphaFold program: (Jumper et al., 2021) p69905 (hemoglobin), p07148 (human t94a variant liver fatty acid binding protein), q9avb0 (n‐acetyl‐d‐glucosamine binding lectin), q9buz4 (traf domain of the tnf receptor‐associated factor 4 protein), p62568 (caerin), p10451 (osteopontin), q6gx35 (translocated actin recruiting phosphoprotein), p49810 (presenilin‐2), and q9m0n8 (gamma‐tuc protein3‐interacting protein 1). The normalized absolute deviation obtained from the KCD method is also included in parenthesis.

**FIGURE 1:**
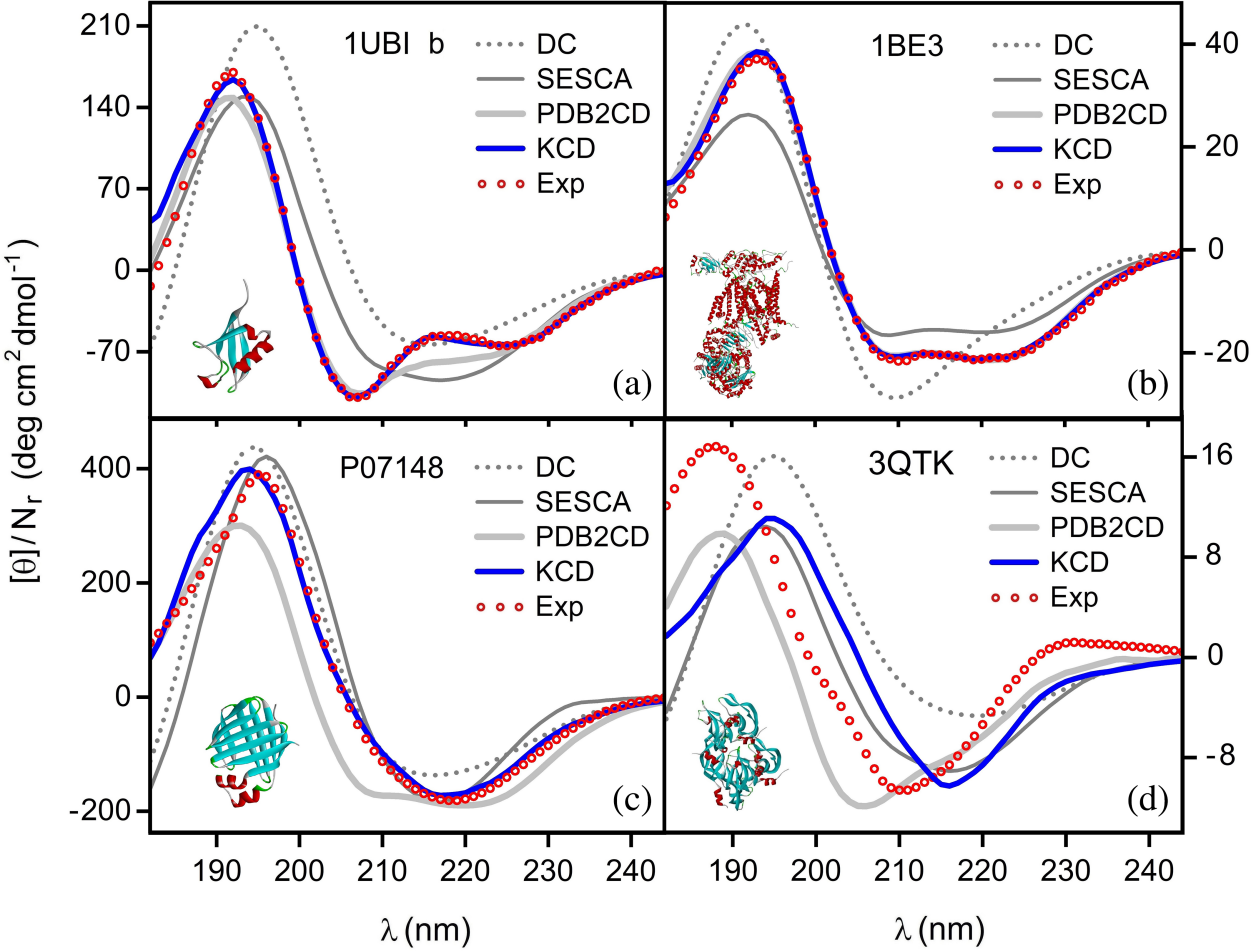
A series of four groups of molar ellipticites per residue $\left[\theta \right]/N_{r}$ plotted as a function of the wavelength λ. Each panel consists of an SRCD experimental spectrum (Ramalli et al., 2022) (Exp, open circles) and the results of four different models: DichroCalc (Bulheller & Hirst, 2009) (DC, dotted line), SESCA (Nagy & Grubmüller, 2021a) (SESCA, thin gray line), PDBMD2CD (Drew & Janes, 2020) (PDB2CD, light gray line), and KCD (KCD, blue line). Each protein is labeled with its corresponding PDB code, except for protein p07148, which is a structural prediction of AlphaFold: (Jumper et al., 2021) (a) 1ubi (ubiquitin, 13.2% α‐helices and 30.3% β‐strands). (b) 1be3 (cytochrome bc1 complex, 42.1% α‐helices and 10.3% β‐strands). (c) p07148 (human t94a variant liver fatty acid binding protein, 9.4% α‐helices and 61.4% β‐strands). (d) 3qtk (vegf‐a, 8.3% α‐helices and 58.3% β‐strands). Of the four proteins, 1ubi belongs to the base set (b) and the three remaining ones are part of the test set.

For instance, in Figure 1 we compare the results obtained within the four models under analysis together with their related experimental SRCD spectra (Ramalli et al., 2022). The proteins under consideration are: 1ubi (ubiquitin), 1be3 (cytochrome bc1 complex), p07148 (human t94a variant liver fatty acid binding protein), and 3qtk (vegf‐a). Only the protein 1ubi belongs to the base set and the other three are part of the test set. As can be observed in Figure 1a–c, there is a good agreement between the molar ellipticities per residue of the KCD model and the corresponding measured data, whereas the spectral curves from the remaining models are farther away from their experimental counterparts. On the other hand, the correct treatment of proteins such as 3qtk is still a challenge for any approach, as can be noticed in Figure 1d. In other respects, a closer inspection of the results of the KCD model in contrast to the measured data reveals the following features. The comparison shown in Figure 1a points out the degree of accuracy attained during the training process of our method. For the protein 1ubi, the corresponding values of $\hat{\Delta }\left[\theta \right]$ for the models DC, SESCA, PDB2CD, and KCD are: 0.66, 0.35, 0.15, 0.02, respectively. In the case of newly predicted spectra, the theoretical curves presented in Figure 1b,c denote fairly good spectral modeling for proteins with a predominant content of *α*‐helices or a symmetrical arrangement of *β*‐strands, respectively. However, the high proportion of disordered *β*‐structures in a protein such as 3qtk decreases the degree of agreement between the calculated and the experimental results, as exhibited in Figure 1d. More in‐depth, the validation of the results obtained by means of the four models under consideration requires a more quantitative assessment. In the specific case of Figure 1b–d, the respective values of $\hat{\Delta }\left[\theta \right]$ for the models DC, SESCA, PDB2CD, and KCD are: 0.31, 0.20, 0.04, 0.03 (1be3); 0.22, 0.24, 0.35, 0.11 (p07148); 0.99, 0.61, 0.52, 0.73 (3qtk).

**FIGURE 2:**
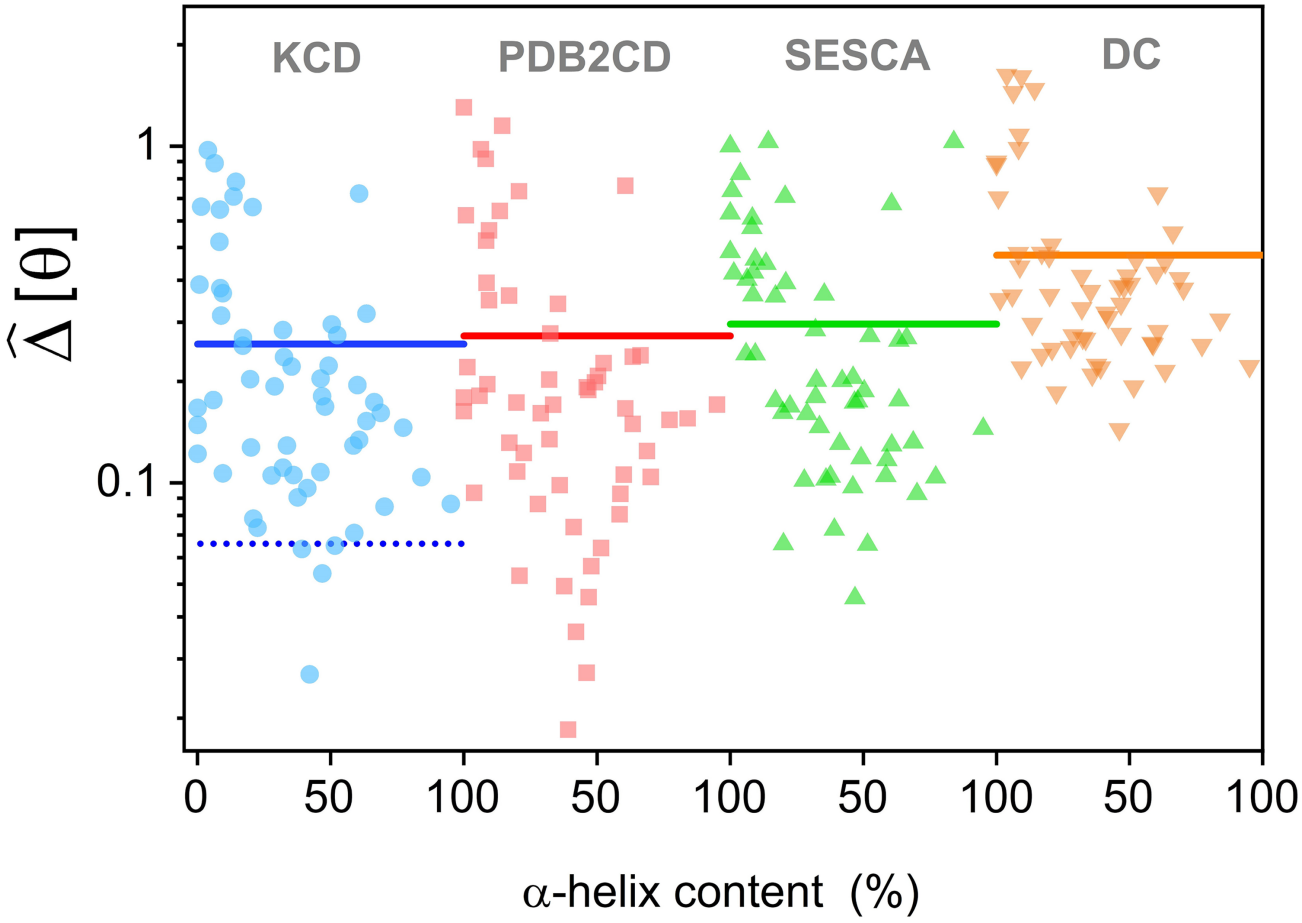
Normalized absolute deviation $\hat{\Delta }\left[\theta \right]$ for a test series of 57 proteins (see text) that are studied through four different methods: KCD (KCD, blue circles), PDBMD2CD (Drew & Janes, 2020) (PDB2CD, red squares), SESCA (Nagy & Grubmüller, 2021a) (SESCA, up green triangles), and DichroCalc (Bulheller & Hirst, 2009) (DC, down orange triangles). All differences are determined with respect to SRCD experimental data (Lees et al., 2006; Ramalli et al., 2022; Whitmore et al., 2017). For each model, the data are plotted as a function of the percentage of α‐helices in each protein. For clarity purposes, the data are shifted between models. The continuous line is the average value $\left\langle \hat{\Delta }\left[\theta \right]\right\rangle$ for each group under analysis. In the case of the KCD method, the average value for the learning set (0.066) is shown in dotted line.

In Figure 2, we present a comparison of the differences $\hat{\Delta }\left[\theta \right]$ between the experimental and the calculated ellipticities for the test set of proteins and including the results of the four models under consideration. Such differences are displayed as a function of the percentage of α‐helix content of each protein and the results for each model are shifted horizontally by 100 units, for clarity purposes. At first glance, there is a general trend in all cases. The degree of agreement between the experimental and the modeled spectra deteriorates (higher NAD values) with a decreasing proportion of α‐helices. In other words, the correct spectral modeling of proteins with a predominant content of coils or *β*‐structures or both may be more difficult, as exemplified in Figure 1d. More remarkably, the average values $\left\langle \hat{\Delta }\left[\theta \right]\right\rangle$ are also plotted in Figure 2 using horizontal lines. For each model, such average values and standard deviations are: 0.26 ± 0.23 KCD, 0.27 ± 0.29 PDB2CD, 0.30 ± 0.24 SESCA, and 0.47 ± 0.36 DC. Although slightly lower, the NAD average value of the KCD model is basically the same as for the PDB2CD model and thus providing some of the best predictions. We want to stress that in the case of the subset defined as structures containing at least 14% of *α*‐helices, the average value for the KCD model becomes 0.19 ± 0.17 (and 0.20 ± 0.21 for the PDB2CD model). On the other side, the DC method gives rise to the least accurate spectra of the four models. In addition to this comparison, some interesting applications of the KCD model are discussed in the Section 3.

### 2.3 Molecular dynamics simulations

We carry out molecular dynamics (MDs) simulations within the GROMACS 2023 package (Abraham et al., 2015) together with the Amber99 force field (Wang et al., 2000) and the TIP4P water model (Jorgensen et al., 1983). Such simulations are performed in the NPT ensemble, using the Berendsen thermostat (Berendsen et al., 1984) and also the Parrinello‐Rahman barostat (Parrinello & Rahman, 1981) at a temperature of 298 K for 50 ns. The leapfrog integrator is utilized with a time step of Δt = 2 fs. The simulations are carried out in a cubic box of side 7.49 nm, periodic boundary conditions, and the short‐range electrostatic and van der Waals cutoffs are fixed at 1 nm. Moreover, the long‐range electrostatics are treated through the particle mesh Ewald method (Ewald, 1921).

## 3 RESULTS

### 3.1 Results of the KCD model

**FIGURE 3:**
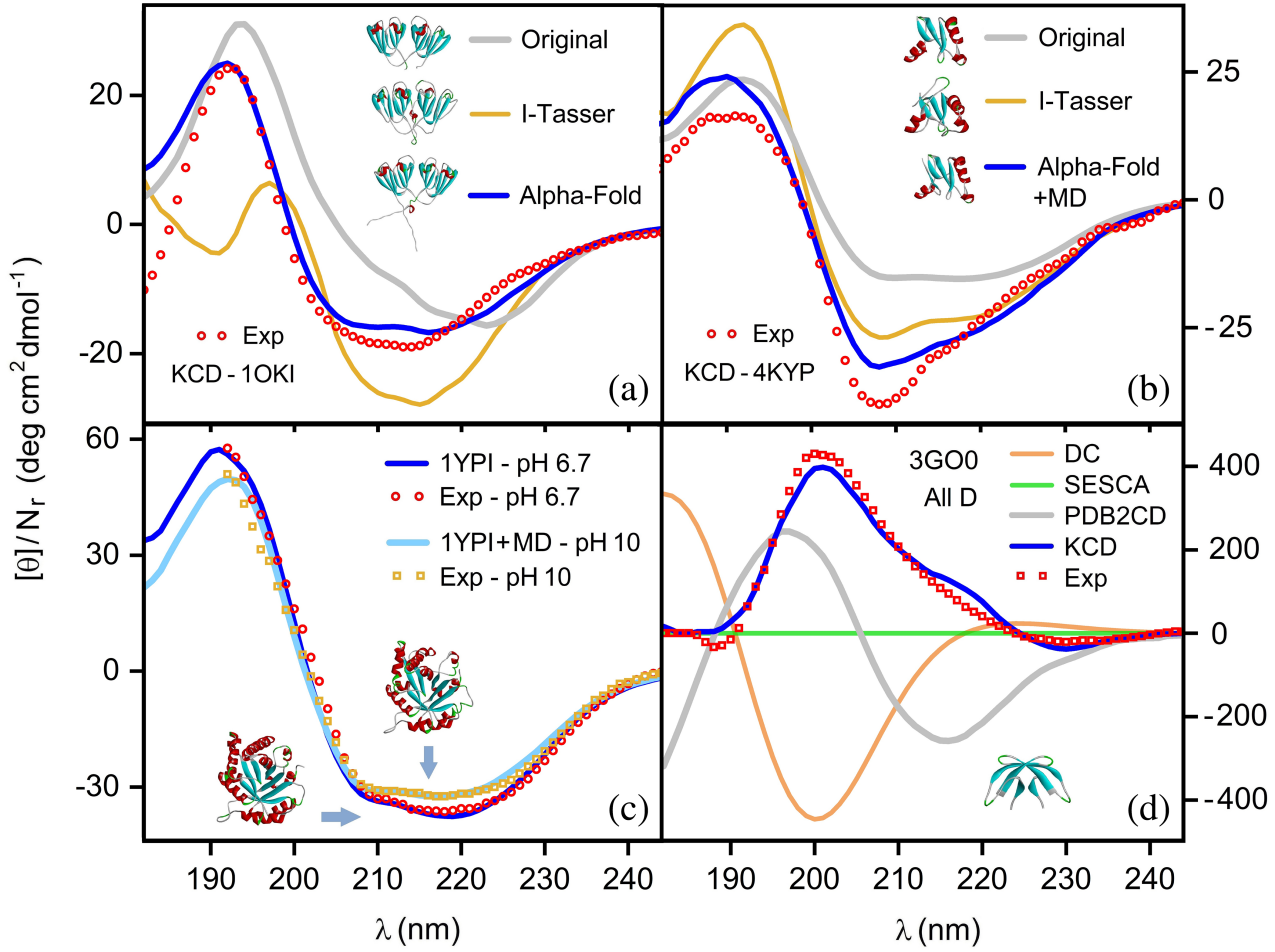
A series of CD spectra in the form of molar ellipticities per residue ($\left[\theta \right]/N_{r}$ as a function of the wavelength λ) that are closely related to the results of the KCD model (KCD). Two reconstruction schemes are compared in panels (a) and (b) for the proteins 1oki (beta‐b1‐crystallin) and 4kyp (beta‐scorpion toxin), respectively. The calculated curves are shown in comparison with their corresponding SRCD spectra (Ramalli et al., 2022) (Exp, open circles). In both cases, the original CD curves (light gray lines) are plotted together with the emerging spectra of proteins that have been reconstructed by means of the algorithms I‐Tasser (Yang & Zhang, 2015) (yellow sienna lines) and AlphaFold (Jumper et al., 2021) (blue lines). In all cases, MD denotes a further stage of molecular dynamics (see text). (c) Effect of a pH change on the secondary structure of the protein 1ypi (yeast triosephosphate isomerase). The continuous lines are the results of the KCD model (blue line—pH 6.7 and cyan line—pH 10). The experimental data are from García‐Gutiérrez et al. (2022) (open circles—pH 6.7 and open squares—pH 10). (d) Measured molar ellipticity of the all D‐protein (All D) 3go0 (D‐enantiomer of human alpha‐defensin 1) (Wei et al., 2009) (open circles) shown in comparison with the theoretical curves from the models: DichroCalc (Bulheller & Hirst, 2009) (DC, orange line), SESCA (Nagy & Grubmüller, 2021a) (SESCA, green line of 0 value), PDBMD2CD (Drew & Janes, 2020) (PDB2CD, light gray line), and KCD (KCD, blue line).

In our previous paper (Granados‐Ramírez & Carbajal‐Tinoco, 2022), we have demonstrated that the KCD approach can be used to describe other conformational changes: by modifying the characteristics of the electrolyte (for the protein s100a12 in the presence of Ca^2+^ or Zn^2+^) and by direct interactions (the complexation of the peptides hr1 and hr2). Here, we revisit three more applications, and we study a new one. For diverse reasons, the PDB structure of a protein may have missing residues with respect to the FASTA sequence. In Figure 3a,b, we examine some reconstruction procedures that are validated by comparing the predictions of the KCD model with the measured spectra. For instance, the crystal structure of protein 1oki (beta‐b1‐crystallin) lacks 53 residues from a total of 420 residues of the FASTA sequence. In Figure 3a, we present the experimental spectrum of protein beta‐b1‐crystallin together with three results of the KCD model, namely, the spectrum of the original protein (incomplete) and the normalized spectra obtained after a reconstruction stage with the algorithms I‐Tasser (Yang & Zhang, 2015) and AlphaFold (Jumper et al., 2021). A similar comparison is carried out in Figure 3b for the protein 4kyp (beta‐scorpion toxin) that has 47 missing residues from the total of 336 residues of the FASTA sequence. An improvement stage consists of a MD simulation that is performed after the AlphaFold reconstruction procedure. The MD stage is also tested in the case of the protein 1oki without any noticeable difference. As can be observed in both panels, the two reconstructions related to AlphaFold are clearly closer to the experimental data. For comparison purposes and using the PDB2CD method, we present the same type of comparison in the Supplementary material section. As it can be observed in the Figure 1 of such section, the PDB2CD approach is only slightly less accurate than the KCD method for the AlphaFold reconstruction of the 1oki protein. On the other hand, the PDB2CD method is basically unable to draw a distinction between the original spectrum and the two spectra obtained from the reconstructed versions of the 4kyp protein, as shown in the Figure 2 of such section.

In Figure 3c, we describe the conformational modification of protein 1ypi (yeast triosephosphate isomerase) by a variation in the pH. A simulation and experimental assessment of such structural change is reported in García‐Gutiérrez et al. (2022). In addition to the information provided by such study, here we present a comparison between the normalized spectroscopic results of the KCD model and the corresponding experimental curves at the pH values of 6.7 and 10. We point out that the structure at pH 10 is obtained from the original conformation by employing the previously described MD algorithm with an estimate for the p$K_{a}$ value given by the PROPKA program (Olsson et al., 2011) that can be found at the APBS‐PDB2PQR web server (Jurrus et al., 2018). The main secondary structural features of the two yeast triosephosphate isomerases are the following: (Frishman & Argos, 1995) 32.6% α‐helices, 15.6% β‐strands, 22.5% coils, 29.5% others (pH 6.7); 27.2% α‐helices, 15.4% β‐strands, 26.8% coils, 30.6% others (pH 10). As can be observed in Figure 3c, there is a noticeable change in both spectral curves and a good accord between the theoretical and the measured data. In this case, the PDB2CD method provides comparable results (not shown).

In recent years, there has been a relevant effort to understand the effects of D‐amino acid residues and all D‐proteins together with regular residues and proteins (Guardiola et al., 2021). For instance, D‐residues have been found in the peptidoglycan chains of bacteria (van Heijenoort, 2001) and thus becoming a possible target for designing new drugs based on peptides or proteins. Here, we mention the capability of the KCD model to describe individual D‐residues as well as full D‐proteins. In Figure 3d, we compare the measured spectrum for the all D‐protein 3go0 (D‐human α‐defensin) (Wei et al., 2009) with the normalized spectral results emerging from the models DC, SESCA, PDB2CD, and KCD. As shown in such figure, the KCD model delivers the CD spectrum that is closest to the experimental curve. The different features that have been considered here are incorporated in a web service.

### 3.2 The KCD web server

**FIGURE 4:**
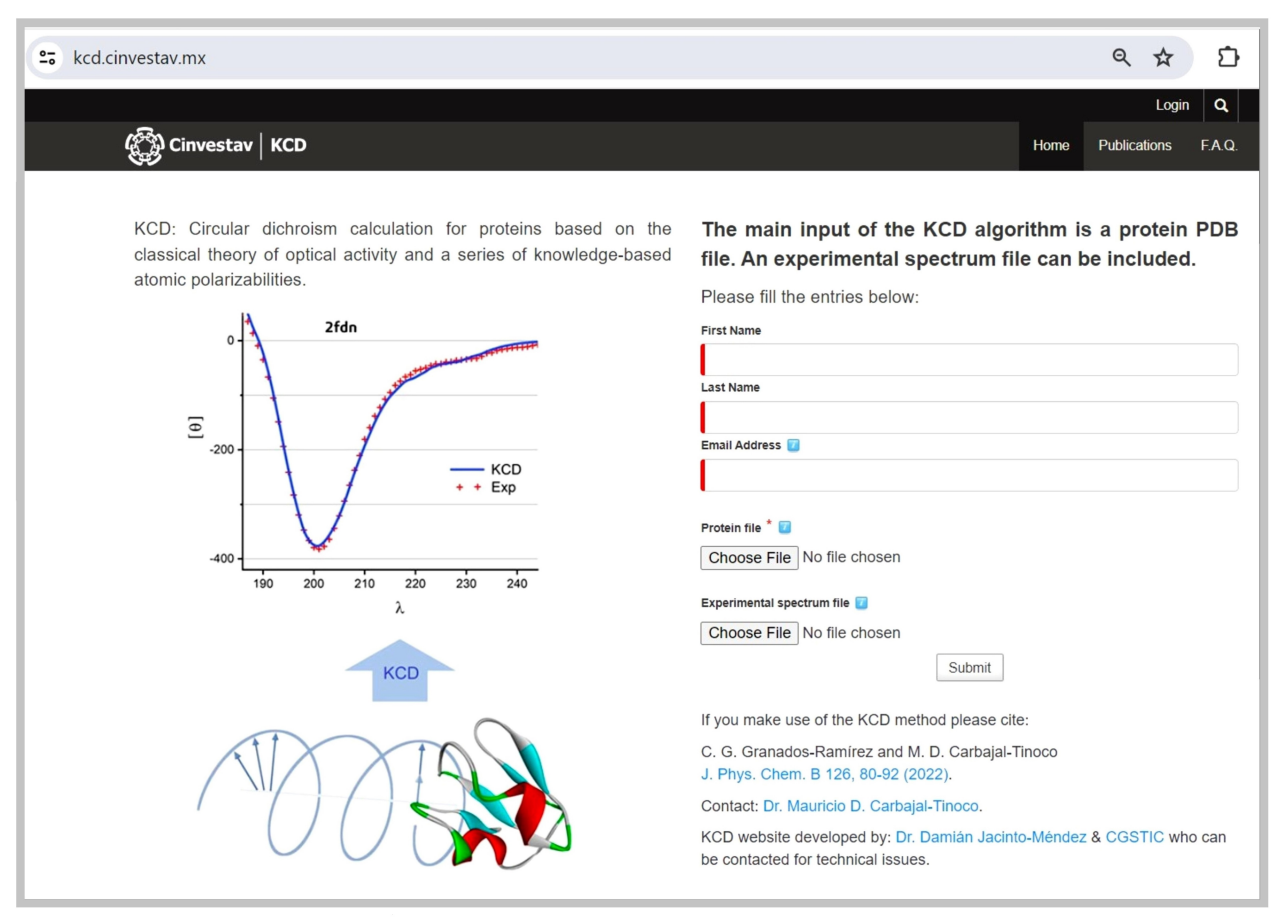
Main input page of the knowledge‐based prediction web server of circular dichroism, KCD. At this point, the user can upload the contact information together with the PDB file of the protein structure as well as an optional file of an experimental CD spectrum.

**FIGURE 5:**
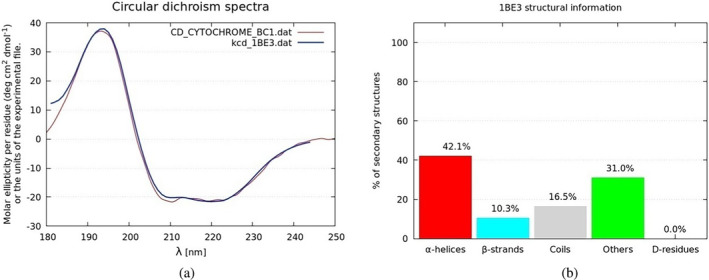
An example of the two graphs sent to the user. A comparison between two CD spectra is displayed in panel (a). The red line corresponds to the experimental spectrum provided by the user (in this case, cytochrome bc1) (Ramalli et al., 2022) and the blue line is the result obtained through the KCD method for the protein 1be3. The bar graph of the panel (b) shows the structural information of protein 1be3, according to the STRIDE algorithm (Frishman & Argos, 1995).

In Figure 4, we present the main input page of the KCD web server, where the user can upload the PDB file of the protein under consideration. An additional spectrum file can be included for both normalization and comparison purposes. Moreover, the user is encouraged to upload an experimental file (as a simple two‐column ASCII file). The front end of the KCD method is a web server (https://kcd.cinvestav.mx/) that manages the data files (.pdb and .dat or a similar extension) together with basic information about the user such as name and email address. The web server works in conjunction with a Python‐C daemon to carry out the next tasks: receive, parse, calculate, and send back the results to the user. In more detail, once a job request is made from the KCD site, a server with a Linux based daemon program is used to initiate the calculation according to this flow: PDB file pretreatment for eventual incorrect entries, read out of the PDB file with the Biopython PDB parser (Cock et al., 2009), and secondary structure assignment by using the STRIDE algorithm (Frishman & Argos, 1995). Such assignment is then utilized to compute the appropriate set of polarizabilities, as previously explained. The molar ellipticity spectrum is determined by utilizing a parallelized C code on CPU cores and the calculated spectrum is normalized with the experimental data, if available. Finally, the following results are sent by email to the user: the ensuing figure of the calculated CD spectrum plotted together with the experimental curve, a bar graph of the secondary structure distribution (in percentage), and the ASCII data file of the computed spectrum. In Figure 5, we include an example of the two plots sent to the user. At this point, the current version of the KCD method is 6.3.5.

## 4 DISCUSSION

In spite of a modest initial development for the spectroscopy of far‐UV CD, during the last two decades, it has benefited from numerous theoretical and experimental improvements. By its own nature (here represented by a vector product), it is a highly selective technique that allows to distinguish subtle variations in the secondary structure of a protein. In this work, we have introduced a web server that is intended to be simple and robust. More importantly, it can be quite useful for the community interested in assessing structural properties of proteins, since it delivers accurate CD spectra from the corresponding 3D conformations. Our KCD method offers a different perspective with respect to other kinds of approaches because it provides some additional capabilities such as the treatment of D‐residues. Moreover, the KCD web server is designed to encourage a more frequent comparison between the experimental and the theoretical spectra, which is relatively scarce in the literature. Worthy to note, we would like to acknowledge the collective effort made for the advancement of this technique. On the other hand, and according to our experience, the ideal accuracy for any model is an average NAD (or NRMSD) of at most 0.1, which is a goal that can be reached by increasing the statistics of our training base.

## 5 CONCLUSION

Given the spatial conformation of a protein, the related CD spectrum can be determined by using the KCD web server that can provide a useful service for the community. The KCD method is based on the classical theory of OA and a series of effective atomic polarizabilities that are obtained from the statistical analysis of quality CD spectra and their corresponding 3D structures.

## AUTHOR CONTRIBUTIONS

**Damián Jacinto‐Méndez:** Software; methodology; writing – review and editing. **Carmen Giovana Granados‐Ramírez:** Conceptualization; validation; writing – review and editing; formal analysis. **Mauricio D. Carbajal‐Tinoco:** Validation; writing – review and editing; formal analysis; funding acquisition; writing – original draft; data curation.

## Supporting information


**Data S1:** Supplementary Information.
