# Supplementary material for: KCD: A prediction web server of knowledge‐based circular dichroism
Source: Protein Sci. 2024 Mar 27;33(4):e4967. doi: 10.1002/pro.4967 (PMC10966356; doi:10.1002/pro.4967)
Supplement: Supplementary file 1 — Data S1: Supplementary Information. [file PRO-33-e4967-s001.pdf]

Supplementary Material

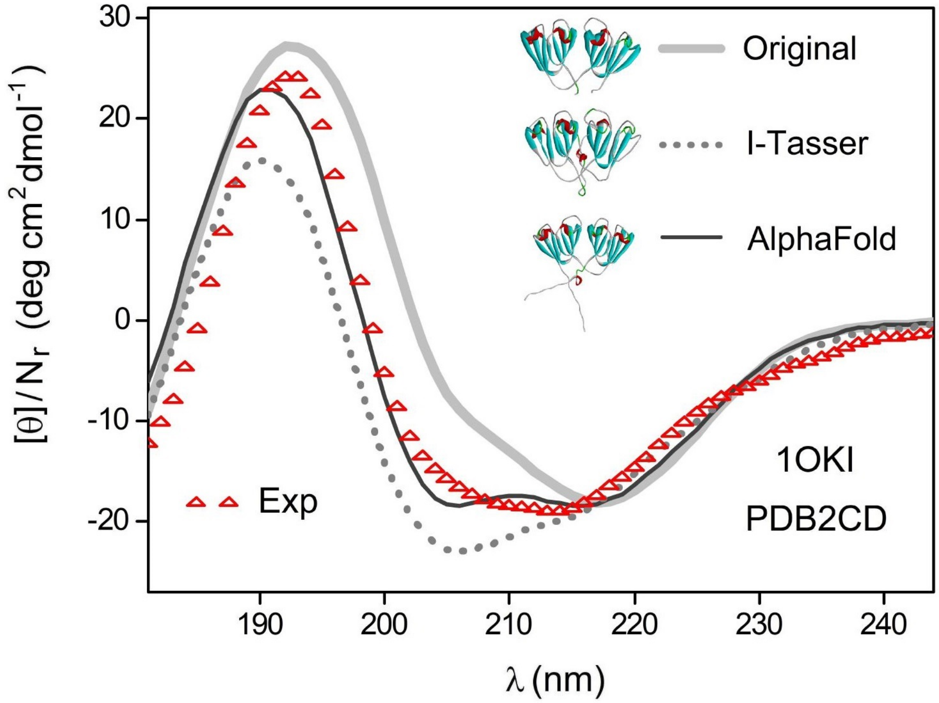

Figure 1: Reconstruction scheme for the protein 1oki (beta-b1-crystallin) using the PDBMD2CD (PDB2CD) method. The theoretical curves are shown together with their corresponding SRCD spectra (Exp, open triangles). The original CD spectrum (light gray line) is plotted along with the reconstructed spectra obtained through the algorithms I-Tasser (dotted line) and AlphaFold (dark gray line). A comparison with the results of the KCD model (shown in parenthesis) is carried out using the normalized absolute deviation, namely: 0.35 (0.55) Original, 0.29 (0.57) I-Tasser, and 0.15 (0.13) AlphaFold (see also Figure 3(a)).

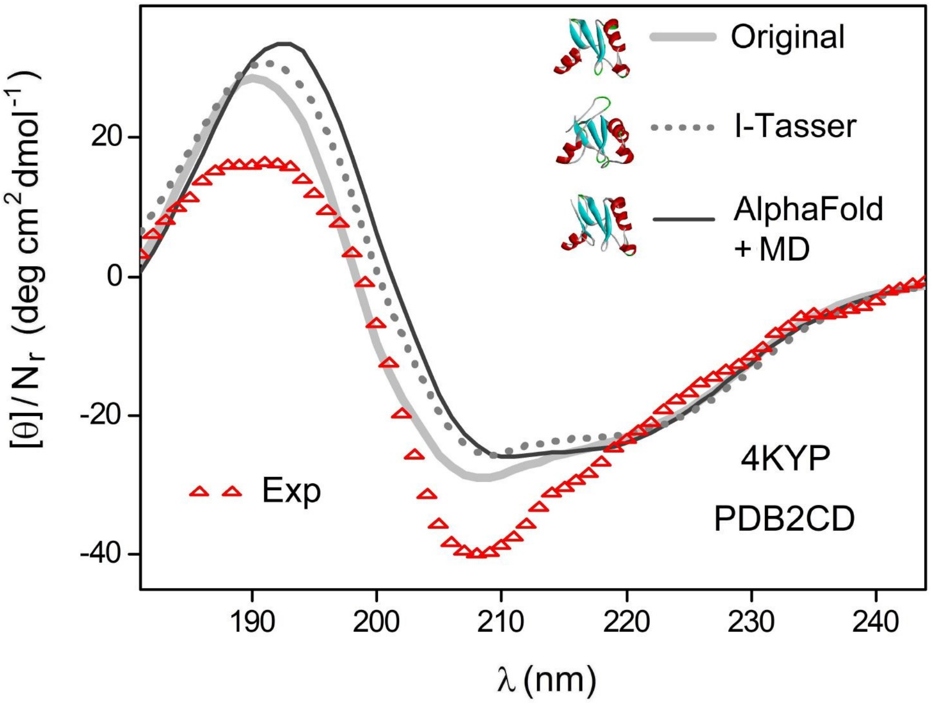

Figure 2: Reconstruction scheme for the protein 4kyp (beta-scorpion toxin) employing the PDBMD2CD (PDB2CD) method. The calculated curves are displayed besides their corresponding SRCD spectra (Exp, open triangles). The original CD spectrum (light gray line) is shown along with the reconstructed spectra obtained through the algorithms I-Tasser (dotted line) and AlphaFold + MD (dark gray line). A comparison with the results of the KCD method (in parenthesis) is established by using the normalized absolute deviation, i.e.: 0.23 (0.49) Original, 0.36 (0.34) I-Tasser, and 0.41 (0.17) AlphaFold + MD (see also Figure 3(b)).
